# 

**Published:** 1876-06

**Authors:** 


### ANNOUNCEMENTS FOR THE MONTH.

**MONDAYS.** SOCIETIES.

*Mondays, June* 5 and 19—Chicago Med. Society, regular meetings at the Washingtonian Home, 8 P. M.

*Mondays, June* 12 and 26 — Chicago Society of Physicians and Surgeons, regular meetings at Grand Pacific, 8 P. M.

CLINICS. *Every Monday.*

At Eye and Ear Infirmary, (Peoria and Adams Sts.) 2 P. M.—Prof. Holmes.

At Central Dispensary (239 W. Van Buren St.), 2 P.M., *Gynecological*—Dr. Adolphus; 3 P.M. *Medical*—Dr. Bridge.

At Mercy Hospital, 2 P. M., *Medical*—Prof. Johnson.

**TUESDAYS.** SOCIETIES.

*Tuesday, June* 13—Academy of Sciences, regular meeting, 8 P. M. (263 Wabash Ave).

CLINICS. *Every Tuesday.*

At County Hospital, 2 P. M., *Medical*—Prof. Lyman; 3 P. M., *Surgical*—Prof. Freer.

At Mercy Hospital, 2 P. M., *Medical*—Prof. Hollister.

**WEDNESDAYS.** CLINICS. *Every Wednesday.*

At County Hospital, 2 P. M., *Ophthalmological*—Dr. Montgomery; 3 P. M., *Gynecological*—Prof. Quine.

At Mercy Hospital, 2 P. M., *Surgical*—Prof. Andrews.

At Central Dispensary, 2 P. M., *Medical*—Dr. Bridge.

At St. Luke's Hospital, commencing at 1.30 P. M., *Surgical*—Dr. Owens.

**THURSDAYS.** CLINICS. *Every Thursday.*

At Central Dispensary, 2 P. M., *Diseases of Chest*—Dr. Ingals.

At Mercy Hospital, 2 P. M., *Medical*—Prof. Johnson.

**FRIDAYS.** SOCIETIES.

*Friday, June* 9—State Microscopical Society of Illinois, regular meeting at the Academy of Sciences, 8 P. M.

CLINICS. *Every Friday.*

At County Hospital, 2 P. M., *Medical*—Prof. Lyman; 3 P. M., *Surgical*—Prof. Freer.

At Mercy Hospital, 2 P. M., *On Diseases of Eye and Ear*—Prof. Jones.

At Central Dispensary, 2 P. M., *Gynecological*—Dr. Adolphus.

**SATURDAYS.** CLINICS. *Every Saturday.*

At Rush College, 2 P. M., *Surgical*—Prof. Gunn; 3 P. M., *Diseases of the Brain and Nervous System*—Dr. Hay.

At Chicago College, 2 P. M., *Surgical*—Prof. Andrews; 3 P. M., *Medical*—Prof. Davis.

☞ At all the above Clinics visiting regular practitioners are, we believe, admitted.

